# Supplementary material for: Identification of photoperiod-induced specific miRNAs in the adrenal glands of Sunite sheep (Ovis aries)
Source: Front Vet Sci. 2022 Jul 22;9:888207. doi: 10.3389/fvets.2022.888207 (PMC9354845; doi:10.3389/fvets.2022.888207)
Supplement: Supplementary file 1 [file Data_Sheet_1.ZIP › Supplementary file/miRDeep2 file.zip!/miRDeep2 file/Know_Structure/LP42A3/oar-mir-181a-2.pdf]

miRBase precursor : oar-mir-181a-2
Total read count : 20527
oar-miR-181a read count : 20291
remaining reads : 236

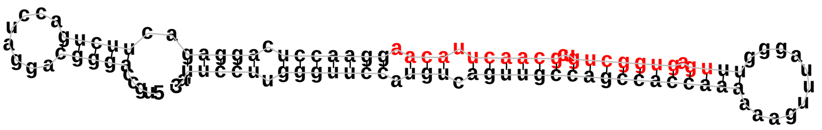

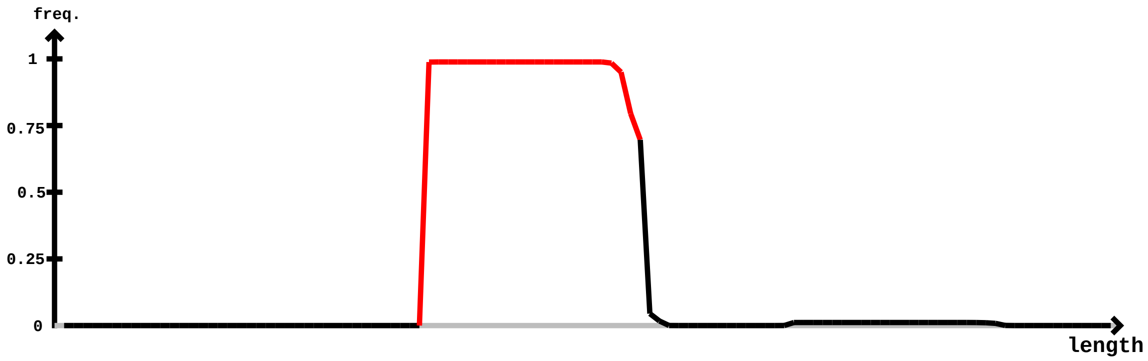

oar-miR-181a

```
5'- ugccagggcaggauccagucuucagaggacuccaaggaacauucaacgcugucggugaguuugggauuugaaaaaaccaccgaccguugacuguaccuuggguuccuua   -3'    exp
    ....(((((........)))))..(((((.(((((((.(((.((((((..(((((((.((((...........))))))))))))))))).))).))))))).))))).    reads      mm          sample
    .....................................aacauucaacgcugucgg......................................................    1          0           seq
    .....................................aacauucaacgcugucggu.....................................................    89         0           seq
    .....................................aacauucaacgcugucggug....................................................    698        0           seq
    .....................................aacauucaacgcugucgguga...................................................    3183       0           seq
    .....................................aacauucaacgcugucggugag..................................................    2022       0           seq
    .....................................aacauucaacgcugucggugagu.................................................    13369      0           seq
    .....................................aacauucaacgcugucggugaguu................................................    569        0           seq
    .....................................aacauucaacgcugucggugaguuu...............................................    346        0           seq
    .....................................aacauucaacgcugucggugaguuug..............................................    4          0           seq
    ......................................acauucaacgcugucggugag..................................................    1          0           seq
    ......................................acauucaacgcugucggugagu.................................................    8          0           seq
    ........................................auucaacgcugucggugagu.................................................    1          0           seq
    ..........................................................................aaccaccgaccguugacugua..............    3          0           seq
    ..........................................................................aaccaccgaccguugacuguac.............    2          0           seq
    ...........................................................................accaccgaccguugacugu...............    4          0           seq
    ...........................................................................accaccgaccguugacugua..............    13         0           seq
    ...........................................................................accaccgaccguugacuguac.............    44         0           seq
    ...........................................................................accaccgaccguugacuguacc............    157        0           seq
    ...........................................................................accaccgaccguugacuguaccu...........    10         0           seq
    ...........................................................................accaccgaccguugacuguaccuu..........    1          0           seq
    ............................................................................ccaccgaccguugacuguac.............    2          0           seq
```
